# Osteogenic Effect of Pregabalin in Human Primary Mesenchymal Stem Cells, Osteoblasts, and Osteosarcoma Cells

**DOI:** 10.3390/life12040496

**Published:** 2022-03-28

**Authors:** Nele Wagener, Pietro Di Fazio, Kai Oliver Böker, Georg Matziolis

**Affiliations:** 1 Department of Trauma Surgery, Orthopedics and Plastic Surgery, University Medical Center Goettingen, Robert-Koch-Str. 40, 37099 Göttingen, Germany; kai.boeker@med.uni-goettingen.de; 2 Department of Visceral Thoracic and Vascular Surgery, Philipps University Marburg, Baldingerstraße, 35043 Marburg, Germany; difazio@med.uni-marburg.de; 3 Orthopaedic Professorship of the University Hospital Jena, Orthopaedic Department Waldkliniken Eisenberg, 07607 Eisenberg, Germany; g.matziolis@waldkliniken-eisenberg.de

**Keywords:** bone defects, osteoblastogenesis, human bone cells, pregabalin, antiepileptic drugs

## Abstract

Seventy million patients worldwide are suffering from epilepsy. The long-term use of antiepileptic drugs causes the alteration of the bone tissue and its metabolism, thus increasing the risk of fractures. Clinical and pre-clinical studies have highlighted conflicting data on the influence of the relatively new antiepileptic drug pregabalin (Lyrica^®^). The objective of the present study was therefore to investigate its cytotoxicity in primary human osteoblasts (hOB). HOB and human mesenchymal stem cells (hMSC) were isolated from patients. The human osteosarcoma cells MG63 were included as established cell line. Cells were incubated with pregabalin at concentrations ranging from 0 to 40 μg/mL. Time-dependent cell proliferation was measured by automatic cell counting, and metabolism was determined by XTT assay and osseous differentiation by alkaline phosphatase (ALP) activity. Histological examinations of calcium deposit were performed with ALP, Alizarin Red, and von Kossa staining. A concentration-dependent increase in the proliferation of hOB and hMSC was observed after treatment with pregabalin. All cells showed a significant increase in cell metabolism. The osteogenic differentiation, confirmed by the increase of calcium deposit, was promoted by the administration of pregabalin. This effect was already significant at the therapeutic plasma concentration of pregabalin (10 μg/mL). In contrast to the other antiepileptic drugs, pregabalin showed no osteocatabolic effects. Conflicting in-vivo data must therefore be attributed to systemic effects of pregabalin.

## 1. Introduction

Currently, about 70 million people worldwide are suffering from epilepsy and the vast majority of them are treated with medication [1]. In addition, the use of antiepileptic drugs has increased continuously in recent years due to other clinical indications [2,3]. Chronic use of antiepileptic drugs can cause disorders of bone metabolism, the decrease of bone mineral density (BMD), impaired cell growth, osteoporosis, osteomalacia, an increased risk of fracture, and catabolism of 25-hydroxycholecalciferol and 1,25-dihydroxycholecalciferol [4,5,6,7,8,9].

A significant reduction of 25-(OH)-D and 1,25-(OH)-D levels may be associated with reduced calcium absorption, secondary hyperparathyroidism, increased bone resorption, and accelerated bone loss [10]. Worrying results regarding bone metabolism have been reported from studies on long-term administration of antiepileptics for the treatment of seizures, various forms of epilepsy, migraine, mood disorders, chronic pain, and bipolar disorders [11,12]. In partiscular, bone loss, associated with long-term treatment, is a gradual unnoticed process, which often remains undiagnosed and untreated [13]. In addition, polytherapy, long-term use, female gender, very young or old age, and genetic predisposition represent the risk-increasing factors of bone loss [14]. Bone density can be pharmacologically affected by cytochrome P450-inducing antiepileptic drugs (EIAEDSs) and non-CYP-450-inducing (CYP-450) antiepileptic drugs (NEIAEDs) [14]. So far, it is known that the fracture risk is increased by CYP-450-inducing antiepileptic drugs such as carbamazepine and valproate [15]. The mechanism of action of pregabalin is very similar to that of gabapentin, both non-CYP450-inducing antiepileptic drugs. Both substances are GABA analogues and belong to the group of newer anticonvulsants, whose mechanism of action is characterized by the inhibition of voltage-gated calcium channels in thalamic neurons [16]. The newer anticonvulsants pregabalin and gabapentin have shown greater therapeutic breadth, with fewer side effects and interactions with the bone tissue than the classical anticonvulsants [16,17]. In-vitro studies on gabapentin have shown osteocatabolic direct effects, thus impairing the human osteoblastogenesis [18]. Pregabalin has shown to delay the consolidation of spinal fusions in male Wistar rats at a higher dosage than the therapeutic range [19]. Also in male patients, therapeutic dosing of pregabalin resulted in a reduction of bone density within two years [20]. It is unclear whether this effect is of systemic origin or whether pregabalin acts directly on the bone cells. The latter is supported by the fact that pregabalin has been detected in relevant concentrations directly in the human bone of patients [21].

Up to date, there have been no in-vitro studies on pregabalin. Therefore, the objective of the present study was, for the first time, to investigate the effects of pregabalin in primary human osteoblasts (hOB), in human mesenchymal stem cells (hMSC), and in MG63 cells as an established cell line.

## 2. Materials and Methods

The experiments were approved by the local ethics committee (Reg. No.: 2020-2019_1-Material). All patients agreed in advance to participate in the study by subscribing to the informed consent. Human mesenchymal stem cells (hMSC) and human osteoblasts (hOB) were collected from the proximal femora of 20 total hip replacement patients aged 65–75 years. HOB were obtained from the cancellous portion of the femoral head and HMSC were isolated from the bone marrow aspirate of the femoral medullary cavity. Cell counting, XTT-assay, and ALP activity measurement were repeated three times under the same conditions, with four parallel tests, so all results are based on *n* = 12 samples. Densitometric quantification of ALP-, von-Kossa, and Alizarin red staining in hOB and hMSC were conducted from 10 sample images for each group.

### 2.1. HMSC Isolation

The bone marrow aspirated obtained from the femoral medullary cavity was washed once with PBS (4 mL) followed by centrifugation (310× *g*; RT) for 10 min, with the supernatant being discarded. The pellet was resuspended in cell culture medium at a ratio of 1:5, followed by Percoll density gradient centrifugation (30 min at 900× *g*) using 20 mL Percoll preparation per 1 mL bone marrow. The cell pellet was suspended in medium and seeded in cell culture flasks. After 14 days, 10^6^ hMSC/mL were used for experiments.

### 2.2. HOB Isolation

Human cancellous bone samples were dissected into approximately 1–3 mm^3^ bone tissue blocks within 2–5 h of collection. The cartilage/soft tissue was completely removed. This was followed by repeated PBS (4 mL) washing until neither fat droplets nor erythrocytes were visible in the supernatant. 10 cancellous bone cubes were placed in a Petri dish (Thermo Scientific™ Nunc™ Petri dish, Thermo Fisher Scientific, Waltham, MA, USA) and mixed with complete cell culture medium. The medium was changed at two-day intervals. After reaching cell confluence, enzymatic detachment of hOB from the bottom of the Petri dish was achieved using 0.25% trypsin in calcium-free PBS, in order to transfer them to cell culture flasks (SARSTEDT AG und Co. KG, Nümbrecht, Germany). After 14 days of cultivation, 10^6^ hOB/mL were used for the experiments.

### 2.3. MG63 Cells

MG63 osteosarcoma cells, derived from a 14-year-old boy, were used as an established osteoblast cell line [22]. The human cell line MG63 was purchased from ATCC (Manassas, VA, USA) and was cultivated according to the supplier’s recommendation in DMEM-F12 (Gibco, Paisley, UK).

### 2.4. Cell Culture Conditions

HMSC were grown in DMEM low glucose (Gibco, Paisley, UK) supplemented with 10% fetal bovine serum, 2% Hepes buffer (Gibco, Paisley, UK), penicillin (100 Units/mL), and streptomycin (100 µg/mL) (Gibco, Paisley, UK) at 37 °C in a humidified atmosphere containing 5% CO_2_. HOB and MG63 cells, were cultured with DMEM-F12 (Gibco, Paisley, UK), with the addition of 10% fetal bovine serum, penicillin (100 Units/mL), and streptomycin (100 µg/mL) (Gibco, Paisley, UK). 0.1 µM Dexamethasone, 200 µM L-ascorbate-2-phosphate, and 10 mM ß-glycerol phosphate were added for osteogenic differentiation to hMSC. For each experiment, cells were seeded in 48-well plates (SARSTEDT AG und Co. KG, Nümbrecht, Germany) at an initial density of 10,000 cells/mL. The medium was changed and pregabalin (Lyrica^®^) was added at concentrations C0 = 0 μg/mL, C1 = 2.5 μg/mL, C2 = 5 μg/mL, C3 = 10 μg/mL, C4 = 20 μg/mL, and C5 = 40 μg/mL at two-day intervals. The concentrations of pregabalin were based on the therapeutic plasma concentration (10 µg/mL), at two and four times the concentration above/below the therapeutic plasma level, respectively [23].

### 2.5. Cell Proliferation

Cell counting of 48-well plate seeded hMSC, hOB, and MG63 cells was performed with 2-day intervals over a period of 14 days. First, the wells were washed with 0.5 mL PBS and trypsinized with 200 μL of 0.25% trypsin in calcium-free PBS at 37 °C. Complete detachment of adherent cells from the bottom of the wells was checked by light microscopy. Cell counting was performed by an automated cell counter (Cellometer Auto T4 Cell Counter, Nexcelcom Bioscience, Lawrence, MA, USA).

### 2.6. Cell Metabolism

An XTT assay was used to quantify the metabolic effect of pregabalin at concentrations of C0–C5 in hMSC, hOB, and MG63 cells. The XTT assay acted as an indicator of metabolic activity of viable cells, which can reduce yellow tetrazolium salt into orange formazan dye. After 96 h of incubation of 10,000 cells/mL in the 48-well plates, 50 μL of XTT labeling mixture (Cell Proliferation Kit II, Roche Molecular Biochemicals, Switzerland) was added to each well. After incubation at 37 °C for two hours, the spectrophotometric absorbance of the samples was measured by using a microplate ELISA reader (FLUOstar OPTIMA, Microplate Reader, BMG LABTECH, Ortenberg, Germany). The spectrophotometric measurement was quantified as absorbance at a wavelength of 450–500 nm against a reference wavelength of 630 nm.

### 2.7. Measurement of Alkaline Phosphatase (ALP) Activity

ALP activity measurement acts as a common indicator of osteoblast differentiation [24]. The measurement was performed after 10 days of incubation, as all cells (hOB, hMSC, MG63) reached their maximum activity at this time. After removal of the medium, the wells were washed with PBS. After the addition of 200 μL of the lysis buffer (5 mL 1 M glycine, pH 9.8; 50 μL 1 M MgCl_2_; 500 μL 1% Nonidet 40; the difference was made up to 50 mL with H_2_O), incubation was performed at 37 °C for 2 h. In the meantime, the substrate solution consisting of 4 mL diethanolamine (5× concentrate) was diluted 1:2.5 in 6 mL of p-Nitrophenyl Phosphate (0.148/10 mL). After preparing a calibration curve, 50 μL of the supernatant was pipetted into a 96-well plate along with 50 μL of the substrate solution. ALP activity was measured photometrically at a wavelength of 405 nm against a standard curve.

### 2.8. Histochemical Stains

In chamber slides (Nunc Lab-Tek II Chamber Slide System, Thermoscientific, Waltham, MA, USA), 8000 hMSC/hOB were seeded and incubated until cell confluence was achieved. The fresh medium was changed and pregabalin was administered every second day. After 21 days of incubation and the achievement of cell confluence, ALP, von Kossa, and Alizarin Red staining were performed. ALP staining acted as an early marker of osteogenic differentiation. The blue staining highlighted the cytoplasma of ALP-positive cells. The culture medium was removed. To fix the cells, the bottom was covered with 37% formaldehyde for 10 min. A total of 1 mL of ALP staining solution was added to the cells for 30 min at 37 °C. The ALP staining buffer consisted of 5 mL dimethylformamide (99.9%), 407 mg MgCl_2_ × 6 mL H_2_O and 1 L 0.1 M Tris buffer. The ALP staining solution was prepared from 0.6 mg true blue salt, 0.1 mg Naphthol AS-MX-phosphate disodium salt and 1 mL ALP staining buffer. Von Kossa staining functioned as evidence of the osteoblast phenotype and enabled the study of calcium incorporation/mineralization of the osteoid formed by the cells. The culture medium was removed and the cells were fixed with 99.8% ethanol for 1 h at −20 °C. The ethanol was removed, and the cells were washed with tap water three times. 3% silver nitrate solution was added to the cells for 30 min and then they were rinsed three times with tap water. 10% Formaldehyde-sodium carbonate solution (Na_2_CO_3_) was added to the cells for 2 min, after which they were rinsed three times with tap water. 10% sodium thiosulfate solution was added to the cells for 5 min, followed by washing three times with tap water. Alizarin Red staining functioned as evidence of advanced osteogenic differentiation. This staining was used to determine the mineralization capacity of the cells. After removal of the culture medium, the cells were fixed with 99.8% ethanol for 1 h at −20 °C. The ethanol was then removed, and the cells washed three times with tap water. Staining was performed with 0.5% Alizarin Red solution for 10 min at room temperature (RT). The cells were then washed twice with tap water and dried for 10 min at RT.

### 2.9. Software and Statistical Analysis

All data were statistically analyzed using GraphPad Prism 9 Software (GraphPad, San Diego, CA, USA). Student’s *t*-test was used to assess the difference between the two groups. Unless otherwise stated, results were shown as mean values with standard deviations, and statistical differences were considered significant when the *p*-value was <0.05. Densitometric quantification of ALP, Alizarin red, and von-Kossa-staining in hMSC, hOB, and MG63 cells was calculated by ImageJ software. Values are given as mean ± standard deviation from 10 sample images for each group. Significance in the difference between C5, C4, C3, C2, C1, vs. C0 was determined by Student’s *t*-test.

## 3. Results

### 3.1. Analysis of Cell Proliferation in Pregabalin Treated hMSC, hOB, and MG63

**Figure 1:**
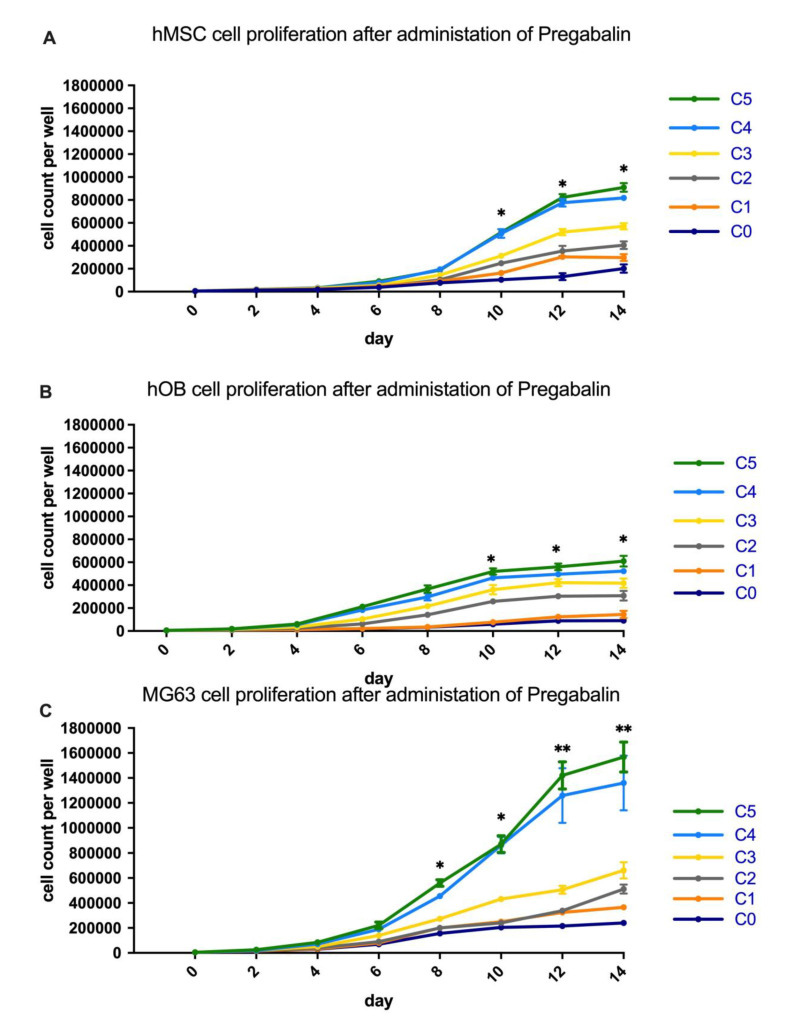
HMSC (**A**), hOB (**B**) and MG63 (**C**) mean cell count per well after treatment with pregabalin at the concentrations 0, 2.5, 5, 10, 20, 40 µg/mL (C0, C1, C2, C3, C4, C5) in the interval of two days. Significance in the difference between C5, C4, C3, C2, C1 vs. C0 was determined by Student’s *t*-test on the 2nd–14th day after seeding. * corresponds to a value of *p* < 0.05. ** corresponds to a value of *p* < 0.01. Values are given as mean ± standard deviation.

The cell counting of hOB, hMSC, and MG63 cells highlighted an increase of cell number after the administration of increasing concentrations of pregabalin (Figure 1A–C). In particular, a statistically significant increase of cell number was observed in hMSC and hOB after 10 days of administration of the 10 µg/mL, 20 µg/mL, and 40 µg/mL (C3–C5) of pregabalin (Figure 1A,B). After 12 and 14 days of administration of 5 µg/mL, 10 µg/mL, 20 µg/mL, and 40 µg/mL (C2–C5) of pregabalin a statistically significant increase of cell number was observed in hMSC and hOB as well.

MG63 cells showed a significant increase of cell number after 8 days of administration of 20 µg/mL and 40 µg/mL (C4–C5) of pregabalin (Figure 1C). After 10 days of administration of 10 µg/mL, 20 µg/mL, and 40 µg/mL (C3–C5) of pregabalin a statistically significant increase of cell number of MG63 cells was observed.

After 12 days of administration of 10 µg/mL (C3) of pregabalin, a statistically significant increase of cell number in MG63 cells was observed, whereas the administration of 20 µg/mL and 40 µg/mL (C4–C5) of pregabalin caused a highly significant increase of cell number. A statistically significant increase of cell number was observed in MG63 cells after 14 days of administration of 5 µg/mL and 10 µg/mL (C2–C3) of pregabalin, whereas the administration of 20 µg/mL and 40 µg/mL (C4–C5) of pregabalin caused a highly significant increase of cell number.

### 3.2. Evaluation of Cell Metabolism after Treatment with Pregabalin

**Figure 2:**
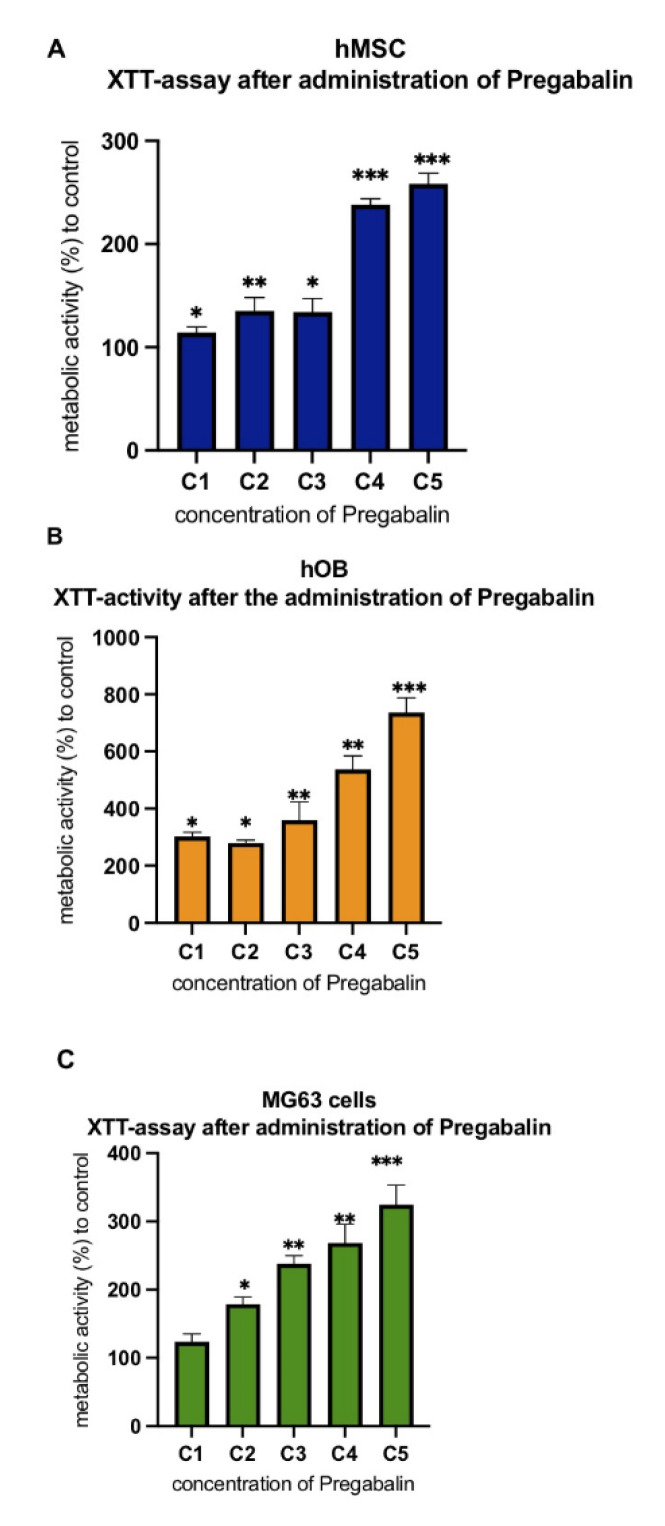
Metabolism of hMSC (**A**), hOB (**B**) and MG63 (**C**) cells after treatment with pregabalin at the concentrations 0, 2.5, 10, 20, 40 µg/mL (C0, C1, C2, C3, C4, C5) was determined by XTT assay. Column height represents XTT metabolism of hMSC, hOB, and MG63 cells depending on the concentration of pregabalin treatment. XTT assay was performed 96 h after cell seeding. Values are given as mean ± standard deviation. Significance in the difference between C5, C4, C3, C2, C1 vs. C0 was determined by Student’s *t*-test. * corresponds to a value of *p* < 0.05. ** corresponds to a value of *p* < 0.01. *** corresponds to a *p*-value of *p* < 0.001.

The analysis of the metabolic active viable cells evidenced that the administration of pregabalin caused a dosage-dependent increase of hMSC, hOB, and MG63 metabolism after 96 h. Pregabalin was not able to increase the metabolism at C1 concentration in MG63 only. Instead, all other concentrations (C2, C3, C4, and C5) were able to increase the cell metabolism in all cells in comparison to untreated cells (Figure 2A–C).

### 3.3. Evaluation of Alkaline Phosphatase Activity in Pregabalin Treated hMSC, hOB, and MG63 Cells

**Figure 3:**
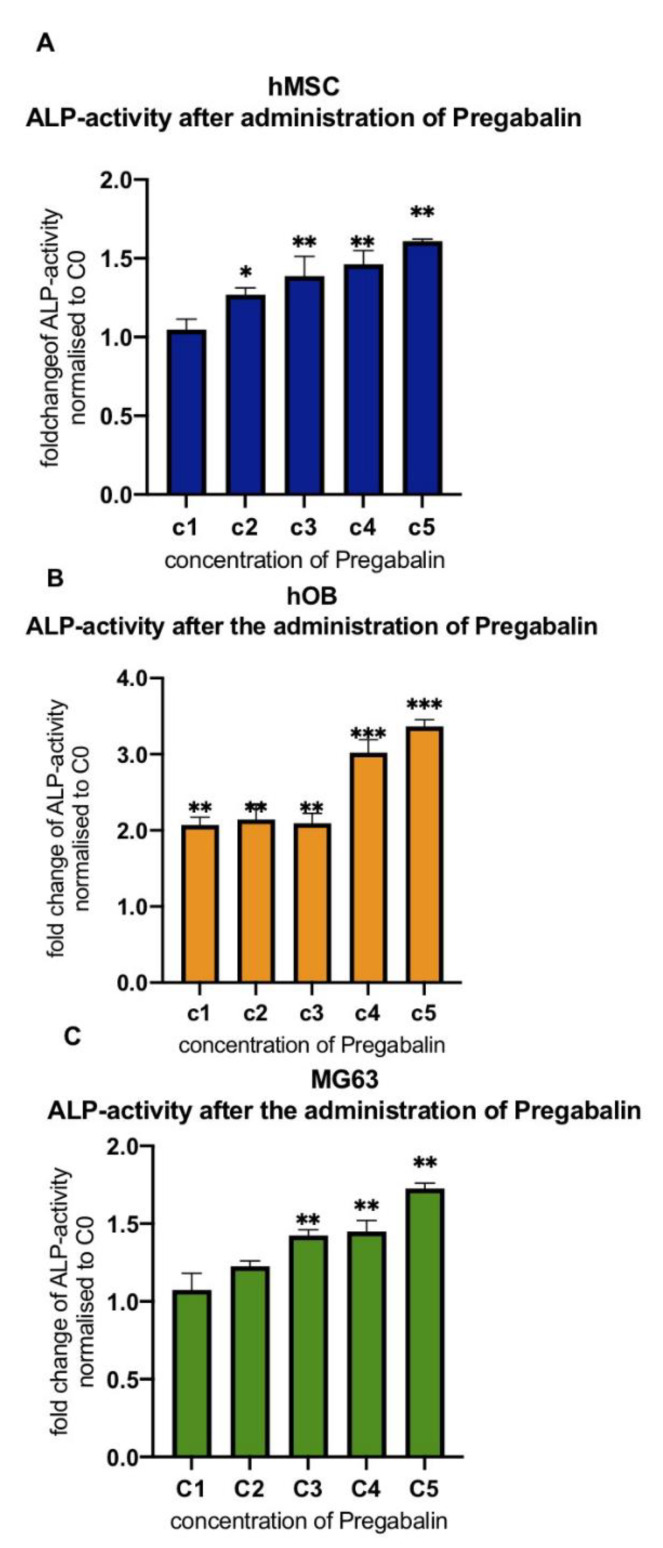
The fold change of ALP activity of pregabalin-treated hMSC (**A**), hOB (**B**) and MG63 (**C**) cells 0, 2.5, 5, 10, 20, 40 µg/mL (C0, C1, C2, C3, C4, C5) relative to untreated cells set at (C0). ALP activity assay was performed on the 10th day after cell seeding. Values are given as mean ± standard deviation. Significance in the difference between C5, C4, C3, C2, C1 vs. C0 was determined by Student’s *t*-test. * corresponds to a value of *p* < 0.05. ** corresponds to a value of *p* < 0.01. *** corresponds to a *p*-value of *p* < 0.001.

The administration of pregabalin, independently of its dosage, caused a significant increase in the activity of alkaline phosphatase in hMSC, hOB, and in MG63 cells. In particular, it was observed that the hOB cells exhibited the highest significant (*** corresponds to a value of *p* < 0.001) ALP-activity (3,1-3,4-fold increase) after 10 days of incubation with 20 and 40 µg/mL (C4–C5) of pregabalin, compared to untreated hOB cells (Figure 3B). HMSC cells showed a similar increase of ALP already observed in hOB cells but with smaller variations compared to untreated cells (Figure 3A). Pregabalin caused a significant (* corresponds to a value of *p* < 0.05) increase of ALP activity in MG63 cells only after the administration of C3, C4, and C5 concentrations (Figure 3C).

### 3.4. Evaluation of Osteogenic Differentiation Markers

**Figure 4:**
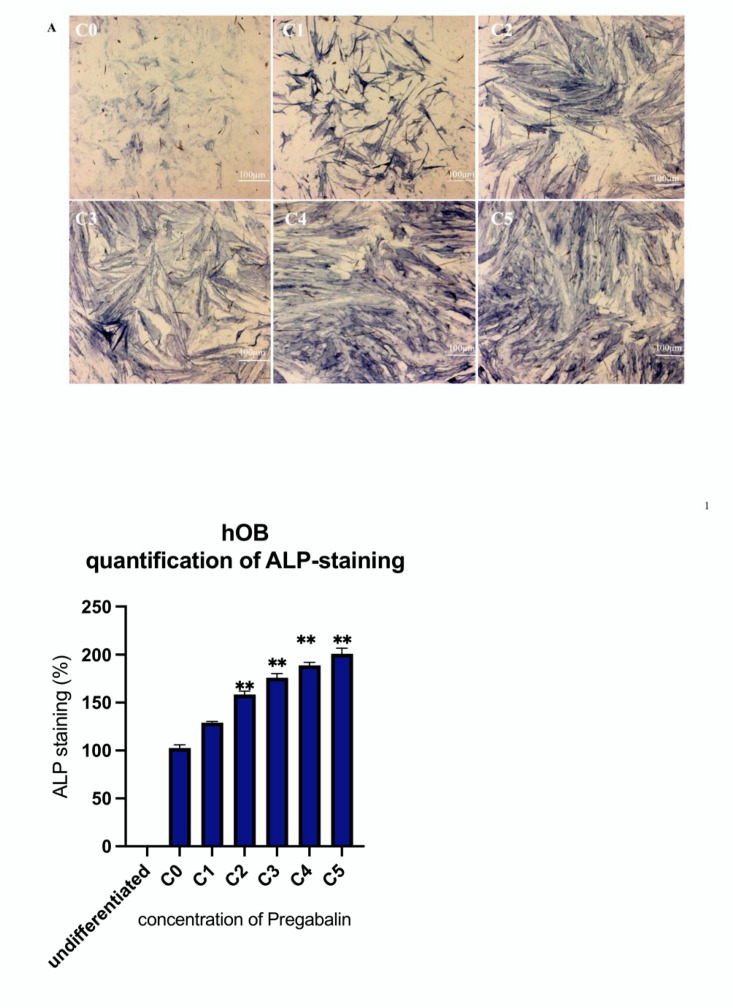

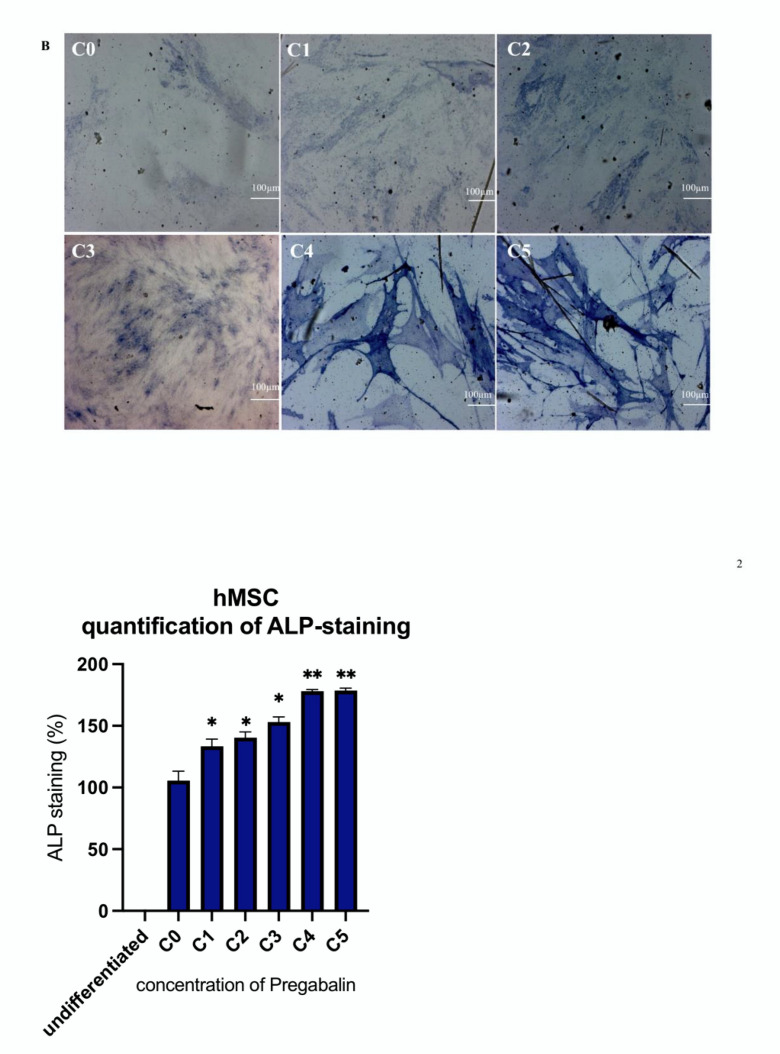
ALP staining of hOB (**A**) and hMSC (**B**) cultured with pregabalin (C1–C5) and without (C0) for 21 days in chamber slides. Treatment with the highest pregabalin concentration enhanced ALP-staining. Densitometric quantification of ALP-staining in hOB and hMSC calculated by ImageJ software. Values are given as mean ± standard deviation from 10 sample images for each group. Significance in the difference between C5, C4, C3, C2, C1 vs. C0 was determined by Student’s *t*-test. * corresponds to a value of *p* < 0.05. ** corresponds to a value of *p* < 0.01.

The administration of increasing concentrations (C1, C2, C3, C4, and C5) of pregabalin caused a significant increase in the intensity of ALP staining (blue), thus sustaining a stronger osteogenic differentiation of hOB (Figure 4A) and hMSC (Figure 4B).

**Figure 5:**
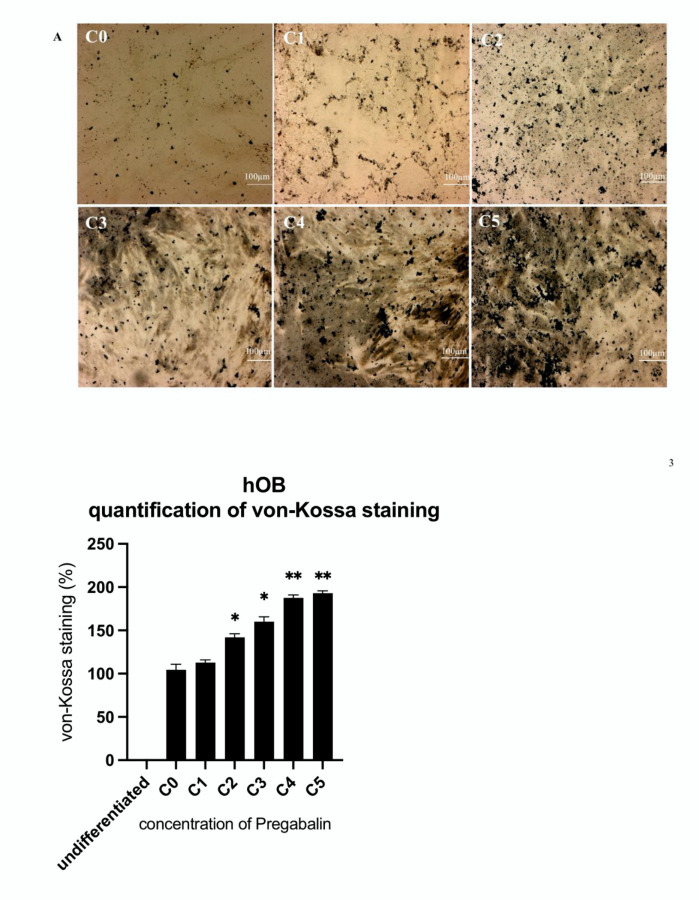

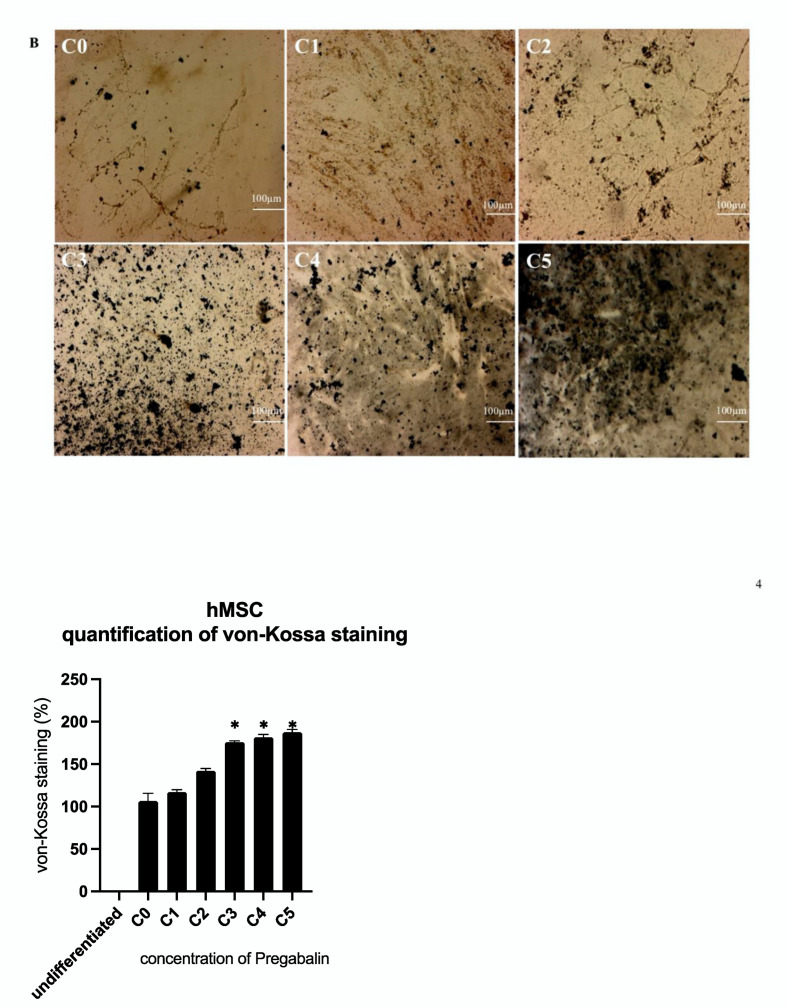
Von-Kossa staining of untreated hOB (C0) (**A**) and hMSC (**B**) treated with pregabalin (C1–C5) for 21 days in chamber slides. Treatment with the highest pregabalin concentration enhanced von-Kossa staining. Densitometric quantification of von-Kossa-staining in hOB and hMSC calculated by ImageJ software. Values are given as mean ± standard deviation from 10 sample images for each group. Significance in the difference between C5, C4, C3, C2, C1 vs. C0 was determined by Student’s *t*-test. * corresponds to a value of *p* < 0.05. ** corresponds to a value of *p* < 0.01.

**Figure 6:**
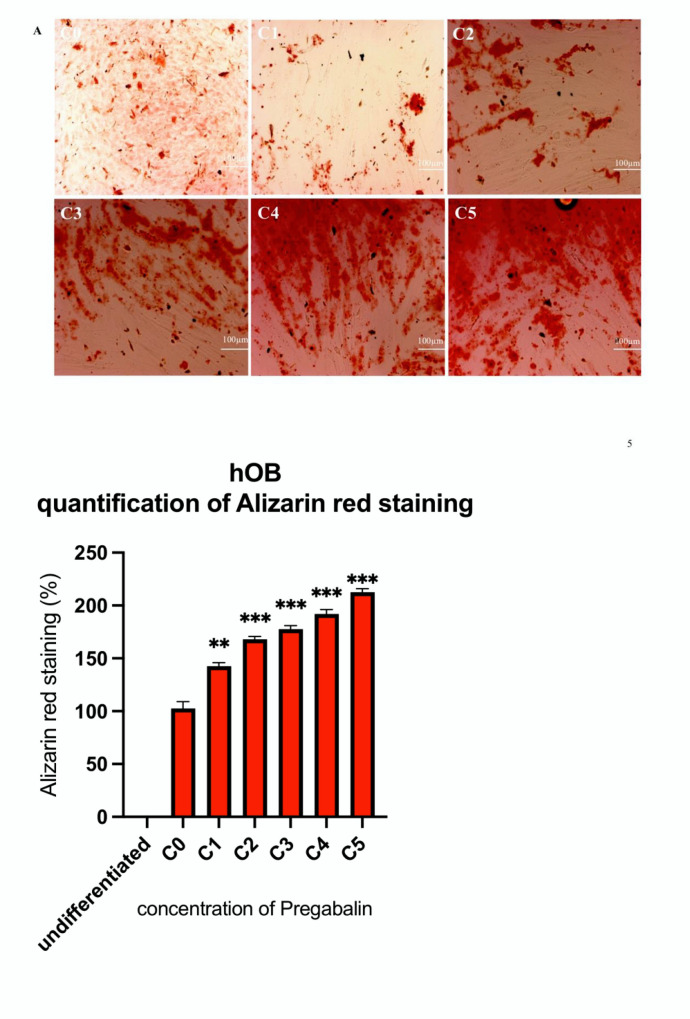

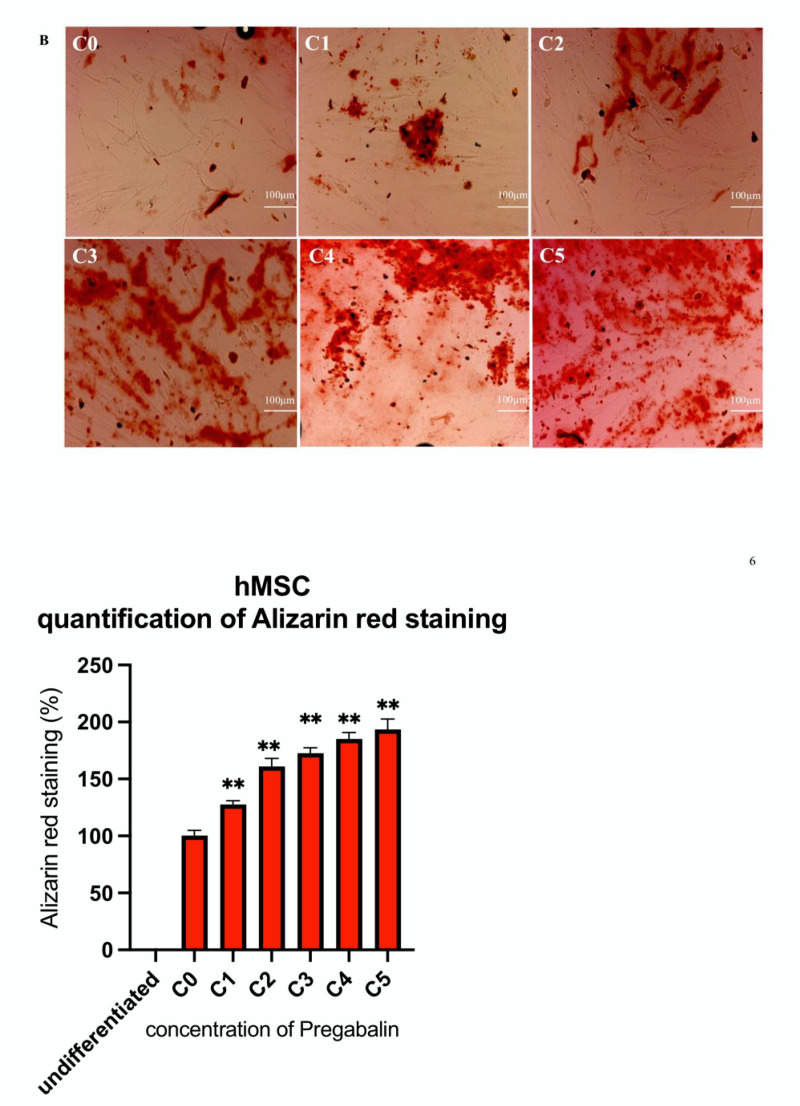
Alizarin red staining of untreated hOB (**A**) and hMSC (**B**) treated with pregabalin (C1–C5) for 21 days in chamber slides. Treatment with the highest pregabalin concentration (C5: 40 µg/mL) enhanced Alizarin Red staining. Densitometric quantification of Alizarin red staining in hOB and hMSC calculated by ImageJ software. Values are given as mean ± standard deviation from 10 sample images for each group. Significance in the difference between C5, C4, C3, C2, C1 vs. C0 was determined by Student’s *t*-test. ** corresponds to a value of *p* < 0.01. *** corresponds to a *p*-value of *p* < 0.001.

The von Kossa staining (black) for the evaluation of calcium mineralization highlighted a significant increase of its intensity, which was proportional to the increased concentration of pregabalin administered to hOB (Figure 5A) and hMSC (Figure 5B). The significant increase of calcium deposit in hMSC and hOB, after the administration of pregabalin, was further confirmed by Alizarin red staining. Once again, the intensity of the staining was proportional to the increased concentration of pregabalin administered in hOB (Figure 6A) and hMSC (Figure 6B).

## 4. Discussion

The main goal of the present study was to investigate the cellular effects of the GABA analogue pregabalin on cell proliferation, cell metabolism, and osteogenic differentiation in primary human mesenchymal stem cells and primary human osteoblasts. The results evidenced that the administration of pregabalin had no osteocatabolic effects in vitro. Its administration caused the increase of proliferation and the metabolism of the cells in a concentration-dependent manner, with at least preserved osteogenic differentiation. The in-vitro dosage-dependent effect of pregabalin was demonstrated here, for the first time, in hMSC, hOB, and MG63 cells. The concentrations of pregabalin were based on the therapeutic plasma concentration (10 µg/mL), at two and four times the concentration above/below the therapeutic plasma level, respectively [23]. Such an effect was observed after staining with ALP, von Kossa, and Alizarin red as well. Over the course of long-term therapy, antiepileptic drug-associated bone damage may be accompanied by secondary osteoporosis, increased bone mass loss, impaired mineralization, and an increased fracture risk [25,26,27,28]. Until now, the cellular effects of the newer antiepileptic drug pregabalin in human primary cells were unknown.

The study by Fernandez-Lopez et al. showed that pregabalin can act directly on bone [21]. Post-mortem analysis by gas chromatography–mass spectrometry detected a level of 90.9% of pregabalin in powdered bone samples after long-term intake in a validation range of 0.3–500 ng/mL. A concentration of 40 ng/mL of pregabalin was detected in bone. Thus, suggesting adverse effects of pregabalin in osteoblasts.

Simko and colleagues have shown that, despite a significant increase of RANKL in the orchidectomized Wistar rats after the administration of pregabalin, those rats accused a significant reduction in bone mineral density [26]. These results suggested that the systemic effects of pregabalin might be gender-related. Analogously to our in-vitro study, Simko and colleagues did not use pregabalin pure drug, which means that it should be considered that the effects observed here might also be attributed to other components included in the commercial medication. Therefore, we could not exclude such effects by the administration of the Lyrica^®^ medication.

A study by Akin and colleagues conducted in 40 patients, 27 women, and 13 men, has found that the administration for six months of pregabalin (Lyrica^®^) in its pharmacological composition had no effects on bone metabolism even independently of gender [20]. Instead, long-term (24 months) intake of Lyrica^®^ caused the reduction of lumbar t- and z-scores.

Another animal study conducted by Imre and colleagues in 32 male Sprague-Dawley rats has shown the influence of pregabalin (Lyrica^®^) [29] in postoperative pain management after lumbar spinal fusion surgery [19]. The results of this study showed an inhibition of spinal fusion formation after the administration of a daily dosage of 30 mg and 100 mg/kg (Body Weight) of pregabalin.

Taken as a whole, the studies on the systemic administration of pregabalin highlight a possible gender-specific osteocatabolic effect on bone metabolism. For the first time, our in-vitro study was able to demonstrate the local cellular effects of pregabalin in hMSC, hOB, and MG63 cells. This study has shown cellular osteoanabolic effects after the administration of increasing concentrations of pregabalin in contrast with the previous studies involving animals treated with a high systemic dosage of Lyrica^®^. Consequently, it can be postulated that the dosage, duration, and mode of administration of pregabalin could have gender-specific systemic effects on bone metabolism. The present study is limited by the fact that only the short-term effects of pregabalin could be replicated, but no long-term effects could be further evaluated.

## 5. Conclusions

The in-vitro findings demonstrated short-time osteoanabolic effects of increasing pregabalin concentration in hMSC, hOB, and MG63 cells.

## Data Availability

Not applicable.

## References

[1] Li H., Zhang Q., Lin Z., Gao F. (2021). Prediction of Epilepsy Based on Tensor Decomposition and Functional Brain Network. Brain Sci..

[2] Druschky K., Bleich S., Grohmann R., Engel R.R., Kleimann A., Stübner S., Greil W., Toto S. (2018). Use and safety of antiepileptic drugs in psychiatric inpatients-data from the AMSP study. Eur. Arch. Psychiatry Clin. Neurosci..

[3] Liu X., Carney P.R., Bussing R., Segal R., Cottler L.B., Winterstein A.G. (2017). Trends in Antiepileptic Drug Use in Children and Adolescents with Epilepsy. Pediatr. Neurol..

[4] Carbone L.D., Johnson K.C., Robbins J., Larson J.C., Curb J.D., Watson K., Gass M., Lacroix A.Z. (2010). Antiepileptic drug use, falls, fractures, and BMD in postmenopausal women: Findings from the women’s health initiative (WHI). J. Bone Miner Res..

[5] Lazzari A.A., Dussault P.M., Thakore-James M., Gagnon D., Baker E., Davis S.A., Houranieh A.M. (2013). Prevention of bone loss and vertebral fractures in patients with chronic epilepsy—Antiepileptic drug and osteoporosis prevention trial. Epilepsia.

[6] Shen C., Chen F., Zhang Y., Guo Y., Ding M. (2014). Association between use of antiepileptic drugs and fracture risk: A systematic review and meta-analysis. Bone.

[7] Collins N., Maher J., Cole M., Baker M., Callaghan N. (1991). A prospective study to evaluate the dose of vitamin D required to correct low 25-hydroxyvitamin D levels, calcium, and alkaline phosphatase in patients at risk of developing antiepileptic drug-induced osteomalacia. Q. J. Med..

[8] Pascussi J.M., Robert A., Nguyen M., Walrant-Debray O., Garabedian M., Martin P., Pineau T., Saric J., Navarro F., Maurel P. (2005). Possible involvement of pregnane X receptor-enhanced CYP24 expression in drug-induced osteomalacia. J. Clin. Investig..

[9] Menon B., Harinarayan C.V. (2010). The effect of anti epileptic drug therapy on serum 25-hydroxyvitamin D and parameters of calcium and bone metabolism—A longitudinal study. Seizure.

[10] Fitzpatrick L.A. (2004). Pathophysiology of bone loss in patients receiving anticonvulsant therapy. Epilepsy Behav..

[11] Petty S.J., Milligan C.J., Todaro M., Richards K.L., Kularathna P.K., Pagel C.N., French C.R., Hill-Yardin E.L., O’Brien T.J., Wark J.D. (2016). The antiepileptic medications carbamazepine and phenytoin inhibit native sodium currents in murine osteoblasts. Epilepsia.

[12] Bialer M. (2012). Why are antiepileptic drugs used for nonepileptic conditions?. Epilepsia.

[13] Nakken K.O., Taubøll E. (2010). Bone loss associated with use of antiepileptic drugs. Expert Opin. Drug Saf..

[14] Siniscalchi A., Murphy S., Cione E., Piro L., Sarro G., Gallelli L. (2020). Antiepileptic Drugs and Bone Health: Current Concepts. Psychopharmacol. Bull..

[15] Hant F.N., Bolster M.B. (2016). Drugs that may harm bone: Mitigating the risk. Cleve Clin. J. Med..

[16] Taylor C.P., Angelotti T., Fauman E. (2007). Pharmacology and mechanism of action of pregabalin: The calcium channel alpha2-delta (alpha2-delta) subunit as a target for antiepileptic drug discovery. Epilepsy Res..

[17] Lee S.K. (2014). Old versus New: Why Do We Need New Antiepileptic Drugs?. J. Epilepsy Res..

[18] Rocha S., Ferraz R., Prudêncio C., Fernandes M.H., Costa-Rodrigues J. (2019). Differential effects of antiepileptic drugs on human bone cells. J. Cell Physiol..

[19] İmre E., Çiftdemir M., Taştekin E. (2020). Effects of pregabalin on spinal fusion. Eur. Spine J..

[20] Akin B., Kelle B., Kozanoglu E. (2021). The Effect of Pregabalin on Bone Metabolism. J. Clin. Densitom..

[21] Fernandez-Lopez L., Mancini R., Pellegrini M., Concetta Rotolo M., Luna A., Falcon M. (2020). Postmortem analysis of quetiapine and pregabalin in human bone. Leg. Med..

[22] White B., Rossi V., Baugher P.J. (2016). Aminolevulinic Acid-Mediated Photodynamic Therapy Causes Cell Death in MG-63 Human Osteosarcoma Cells. Photomed. Laser Surg..

[23] Bockbrader H.N., Radulovic L.L., Posvar E.L., Strand J.C., Alvey C.W., Busch J.A., Randinitis E.J., Corrigan B.W., Haig G.M., Boyd R.A. (2010). Clinical pharmacokinetics of pregabalin in healthy volunteers. J. Clin. Pharmacol..

[24] Rodan G.A., Heath J.K., Yoon K., Noda M., Rodan S.B. (1988). Diversity of the osteoblastic phenotype. Ciba Found Symp..

[25] Ko A., Kong J., Samadov F., Mukhamedov A., Kim Y.M., Lee Y.J., Nam S.O. (2020). Bone health in pediatric patients with neurological disorders. Ann. Pediatr. Endocrinol. Metab..

[26] Simko J., Karesova I., Kremlacek J., Eva Z., Horacek J., Fekete S., Malakova J., Zivna H., Palicka V. (2019). The effect of gabapentin and pregabalin on bone turnover and bone strength: A prospective study in Wistar rats. Pharmacol. Rep..

[27] El-Haggar S.M., Mostafa T.M., Allah H.M.S., Akef G.H. (2018). Levetiracetam and lamotrigine effects as mono- and polytherapy on bone mineral density in epileptic patients. Arq Neuropsiquiatr..

[28] Lee R.H., Lyles K.W., Colón-Emeric C. (2010). A review of the effect of anticonvulsant medications on bone mineral density and fracture risk. Am. J. Geriatr. Pharmacother..

[29] European Medicines Agency (EMA) 2021. Lyrica. 1995–2022 European Medicines Agency. https://www.ema.europa.eu/en/medicines/human/EPAR/lyrica.

